# Dissecting Individual Interactions between Pathogenic and Commensal Bacteria within a Multispecies Gut Microbial Community

**DOI:** 10.1128/mSphere.00013-21

**Published:** 2021-03-24

**Authors:** Jack Hassall, Jeffrey K. J. Cheng, Meera Unnikrishnan

**Affiliations:** a Warwick Medical School, University of Warwick, Coventry, United Kingdom; University of Michigan-Ann Arbor

**Keywords:** *C. difficile*, gut microbiota, interbacterial interactions, mixed biofilms, pathogen-commensal interactions

## Abstract

Interactions of commensal bacteria within the gut microbiota and with invading pathogens are critical in determining the outcome of an infection. While murine studies have been valuable, we lack *in vitro* models to monitor community responses to pathogens at a single-species level. We have developed a multispecies community of nine representative gut species cultured together as a mixed biofilm and tracked numbers of individual species over time using a quantitative PCR (qPCR)-based approach. Introduction of the major nosocomial gut pathogen, Clostridioides difficile, to this community resulted in increased adhesion of commensals and inhibition of C. difficile multiplication. Interestingly, we observed an increase in individual *Bacteroides* species accompanying the inhibition of C. difficile. Furthermore, Bacteroides dorei reduced C. difficile growth within biofilms, suggesting a role for *Bacteroides* spp. in prevention of C. difficile colonization. We report here an *in vitro* tool with excellent applications for investigating bacterial interactions within a complex community.

**IMPORTANCE** Studying interactions between bacterial species that reside in the human gut is crucial for gaining a better insight into how they provide protection from pathogen colonization. *In vitro* models of multispecies bacterial communities wherein behaviors of single species can be accurately tracked are key to such studies. Here, we have developed a synthetic, trackable, gut microbiota community which reduces growth of the human gut pathogen Clostridioides difficile. We report that *Bacteroides* spp. within this community respond by multiplying in the presence of this pathogen, resulting in reduction of C. difficile growth. Defined *in vitro* communities that can be tailored to include different species are well suited to functional genomic approaches and are valuable tools for understanding interbacterial interactions.

## INTRODUCTION

The gut microbiota, which is the largest microbial community found in the human body, plays a key role in an array of essential physiological processes, including immune function, metabolism, and nutrient absorption. Imbalances and shifts in the gut microbiota composition have been associated with multiple conditions including chronic gastrointestinal diseases like inflammatory bowel disease (IBD) (1, 2) and systemic metabolic diseases like diabetes and obesity (3–6). Most studies linking disease states to the microbiome are based on 16S rRNA or whole-microbial-genome sequencing, although recent studies have begun to demonstrate several interesting mechanisms underlying microbiota functions (7). An important function of the gut microbiota is to form a protective barrier against colonization by gastrointestinal pathogens, a property described as colonization resistance (8, 9).

Colonization resistance occurs through an array of direct or indirect bacterial and host interactions including competition for nutrients, host metabolites, and physical space (10). An example of nutrient competition is the commensal Bacteroides thetaiotaomicron, which consumes carbohydrates essential to murine pathogen Citrobacter rodentium, causing it to be excluded (11). Secreted compounds released by the microbiota such as the antimicrobial peptides, bacteriocins, and short-chain fatty acids (SCFAs) can directly affect an invading pathogen. *Bacteroides* spp. were shown to inhibit Salmonella enterica serovar Typhimurium through the production of the SCFA propionate (12). SCFAs also impact epithelial barrier function by affecting production of host molecules including antimicrobial peptides and epithelial mucins (13, 14). Disturbed microbiota and the loss of colonization resistance are associated with several pathogen infections including those by Clostridioides difficile (8), enterohemorrhagic Escherichia coli (15), and Campylobacter jejuni (16).

In the case of C. difficile, a leading cause of health care-associated diarrhea worldwide, colonization occurs only when the microbiota is altered, usually due to treatment with antibiotics such as fluoroquinolones (17). The increased susceptibility to C. difficile infection (CDI) after antibiotic-induced dysbiosis of the gut microbiota is well documented (18, 19). While most studies demonstrating the link between CDI and antibiotic therapy are based on changes in microbial populations by microbiota sequencing, recent studies have reported mechanisms by which the microbiota can prevent C. difficile infections (20). The microbiota in a healthy state consumes or converts primary bile acid into secondary bile acids, reducing the ability of C. difficile to germinate (21). Secondary bile acids such as deoxycholic acid (DCA) and lithocholic acid (LCA) are toxic to vegetative C. difficile (22, 23). Additionally, gut bacteria like Clostridium scindens which encode secondary bile acid synthesis enzymes have been associated with resistance to C. difficile infection (24). The microbiota not only competes for resources but actively inhibits C. difficile through production of bacteriocins such as the thuricin CD, produced by Bacteroides thuringiensis (25).

While the microbiota is clearly important in preventing infections, current knowledge is mainly based on microbiota profiles from feces. The gut microbiota is composed of bacteria within the lumen, which are usually detected in feces, and bacteria associated with the gut mucosa. Few studies have profiled the adherent microbiota population of healthy human guts as they require invasive biopsies (26). While not much is known about the composition and dynamics of this population, it can be viewed as a mixed biofilm community that is closely associated with mucus layers in the gut, which provide a spatial and metabolic niche for the bacteria (27, 28). The understanding of how individual bacteria within such complex communities interact remains poor. *In vitro* systems that mimic gut microbial communities and that are easily trackable are necessary to study interbacterial interactions.

Identifying and quantifying species in a mixed community are challenging; simple microscopy cannot be used as cells are often morphologically too similar. Selective media have proven to be successful with small communities; however, the difficulty of finding species-specific media increases as the community gets larger (29). Quantitative PCR (qPCR)-based approaches have been shown to be successful at predicting cell concentration and biomass formation of individual species in mixed populations (30–32). In this study, we create a representative adherent multispecies gut community, in which we can track behaviors of individual species over time using a qPCR-based method. We have employed this system to study how individual commensal species behave in the presence of the human pathogen C. difficile. We report an increase in *Bacteroides* spp. within this complex biofilm community and a direct impact of a *Bacteroides* species on the growth of C. difficile.

## RESULTS

### Developing a mixed biofilm community and optimization of a propidium monoazide (PMA)-qPCR method for tracking individual species.

In order to develop a complex mixed biofilm community comprising representative gut species, we selected a total of nine gut commensal species (Table 1), based on previous literature describing species present in healthy human gut microbiota (3, 33–35). The species were selected based on high relative abundance, presence across multiple regions (Europe, America, and Asia), and the availability of a sequenced genome. *Firmicutes* and *Bacteroidetes* were overrepresented as these genera are the dominant phylum in the gut (36). These nine species were cultured together as a mixed biofilm as described in Materials and Methods.

**TABLE 1:** List of representative species used to construct a gut microbial community

Species	Phylum	Source^*a*^
Bacteroides dorei (CL02T00C15)	*Bacteroidetes*	BEI Resources
Bacteroides fragilis (3_1_12)	*Bacteroidetes*	BEI Resources
Bacteroides ovatus (3_8_47FAA)	*Bacteroidetes*	BEI Resources
Bacteroides thetaiotaomicron (VPI-5482)	*Bacteroidetes*	Anne Marie Krachler
Bifidobacterium adolescentis (L2-32)	*Actinobacteria*	BEI Resources
Blautia hansenii (20583)	*Firmicutes*	DSMZ
Clostridioides difficile (R20291)	*Firmicutes*	Trevor Lawley
Escherichia coli (83972)	*Proteobacteria*	BEI Resources
Eubacterium hallii (3353)	*Firmicutes*	DSMZ
Faecalibacterium prausnitzii (17677 A2-165)	*Firmicutes*	DSMZ
Ruminococcus gnavus (CC55_001C)	*Firmicutes*	BEI Resources

a DSMZ, Deutsche Sammlung von Mikroorganismen und Zellkulturen; BEI, Biodefense and Emerging Infections Research Resources Repository.

In order to track individual species, primers were designed to target either the topoisomerase I (*topI*) or DNA gyrase subunit A (*gyrA*) region of each strain. These genes were chosen because unlike the conventional 16S gene, they have a single copy number within the genome which improves the accuracy of assumptions made in converting DNA mass to bacterial number. For qPCR-based quantification to be successful, primers have to be highly specific, with preferably no off-target amplification. To test specificity, we tested genomic DNA obtained from each species against primers specific to all the species. We found high levels of specificity with no cross-reacting bands outside the correct lane (see Fig. S1 in the supplemental material).

10.1128/mSphere.00013-21.1 FIG S1 Primer specificity matrix. PCRs were conducted with genomic DNA from each individual species to confirm primer specificity. These products were analyzed on a 2% agarose gel, with a product size between 100 and 150 bp depending on the species. Download FIG S1, PDF file, 2.3 MB. Copyright © 2021 Hassall et al. 2021 Hassall et al. https://creativecommons.org/licenses/by/4.0/ This content is distributed under the terms of the Creative Commons Attribution 4.0 International license.

To convert the qPCR cycle threshold (*C_T_*) values to bacterial numbers, primer efficiencies were first determined for each species with single primer-specific genomic DNA (Table S1A). To rule out nonspecific effects of DNA from other species on primer efficiency, primer efficiencies were determined for each primer set with primer-specific genomic DNA mixed with equal amounts of DNA from the eight other bacterial species (Fig. S2, Table S1B). A primer efficiency between 90 and 110%, which is generally accepted as good enough for accurate qPCR (37), was obtained for all the primers. The mixed DNA standard curves were used for calculating bacterial numbers. *C_T_* values were converted into DNA mass and then into a bacterial number as described in Materials and Methods.

10.1128/mSphere.00013-21.2 FIG S2 Standard curves of the nine commensal bacteria, C. difficile, and B. fragilis. DNA was extracted from exponentially grown cultures of each species and quantified, and dilutions of equal concentration for each species were mixed to generate one sample of mixed genomic DNA. The primer efficiency was determined via qPCR and analyzed with a semilog regression fitted onto the data. Download FIG S2, PDF file, 0.3 MB. Copyright © 2021 Hassall et al. 2021 Hassall et al. https://creativecommons.org/licenses/by/4.0/ This content is distributed under the terms of the Creative Commons Attribution 4.0 International license.

10.1128/mSphere.00013-21.9 TABLE S1 Linearity and primer efficiency of standard curves with single-species bacterial DNA (A) and mixed bacterial DNA (B). Semilog regressions were fitted to each of the standard curves; primer efficiency was calculated from the gradient of these lines. Download Table S1, PDF file, 0.04 MB. Copyright © 2021 Hassall et al. 2021 Hassall et al. https://creativecommons.org/licenses/by/4.0/ This content is distributed under the terms of the Creative Commons Attribution 4.0 International license.

**FIG 1:**
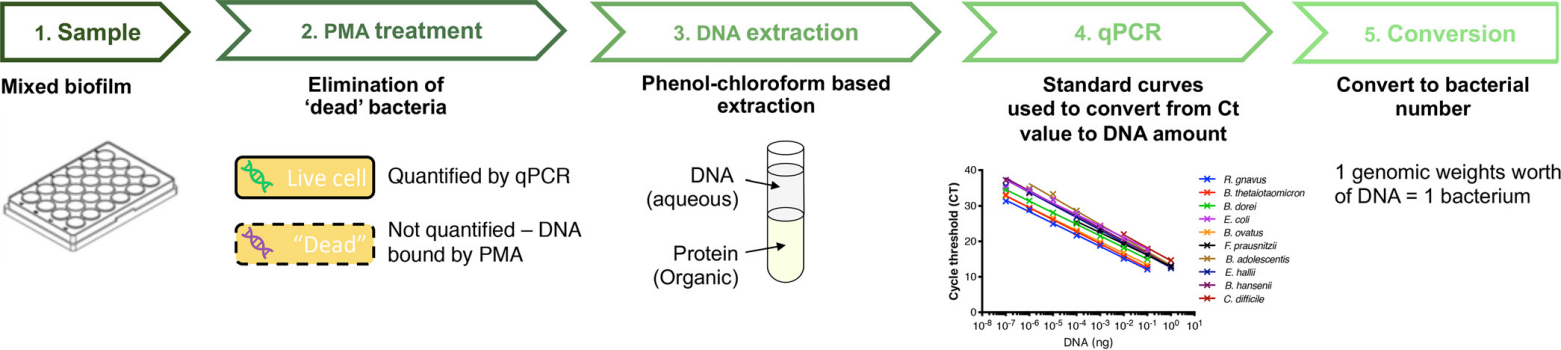
Schematic diagram of the pipeline used to quantify individual species in a mixed biofilm. Samples are PMA (propidium monoazide) treated to ensure quantification of genomic DNA only from live cells. After treatment, cells are lysed, and DNA is extracted, followed by qPCR quantification of DNA and subsequent conversion to bacterial numbers.

For accurate quantification of bacteria, it is essential to quantify only active/live cells. Standard qPCR quantifies all cells, “dead” and “active” alike, which gives an inaccurate abundance of individual species within a population. To improve the accuracy of our quantification, we used propidium monoazide (PMA) to prevent the counting of “dead” bacteria, i.e., those with compromised membranes. PMA is a photoactivated DNA binding dye that can target only cells with ruptured membranes or “dead” cells (38–41). When PMA is photoactivated, it covalently binds to double-stranded DNA (dsDNA), cross-linking the two strands, and this binding prevents DNA amplification during PCR (31). Hence, biofilm samples were first treated with an optimized concentration of PMA, followed by genomic DNA extraction and then analysis by qPCR (Fig. 1). Single-species biofilms treated with PMA resulted in a decrease in bacterial numbers (Fig. S3A and B), and biofilm formation by each of the nine bacterial species was followed over time (Fig. S3C). The decrease in bacterial numbers measured was higher in 48-h biofilms, which was expected, as there are likely more dead cells in biofilms at later times.

10.1128/mSphere.00013-21.3 FIG S3 (A and B) The effects of PMA treatment on bacterial numbers within a single-species biofilm at 24 h (A) and 48 h (B). Control biofilms were treated with water instead of PMA. (C) A comparison of the PMA-treated single-species biofilms over time. Data shown are the means from a technical triplicate. (D) A comparison between qPCR-predicted bacterial numbers and bacterial numbers obtained from plating (CFU assay). Biofilms of each species were grown for 24 h; these were then washed and resuspended in 1 ml PBS. Fifty microliters of each sample was taken for serial dilution and plating; the rest was used in the PMA-qPCR pipeline. Data shown represent the means from three independent experiments in triplicate. A two-way ANOVA showed no significant difference between plating and the qPCR technique. Download FIG S3, PDF file, 0.2 MB. Copyright © 2021 Hassall et al. 2021 Hassall et al. https://creativecommons.org/licenses/by/4.0/ This content is distributed under the terms of the Creative Commons Attribution 4.0 International license.

To confirm the accuracy of this qPCR-based quantitation assay, we grew single-species biofilms and tested the prediction bacterial number from qPCR versus the actual CFU values obtained from plating (Fig. S3D). We chose a species representative of each phylum (*Firmicutes*, *Bacteroidetes*, *Proteobacteria*, and *Actinobacteria*). We found that the predicted values are similar to that of the CFU assay; a two-way analysis of variance (ANOVA) predicts there is no significant difference between the two techniques.

### Tracking individual species within a microbiota community.

**FIG 2:**
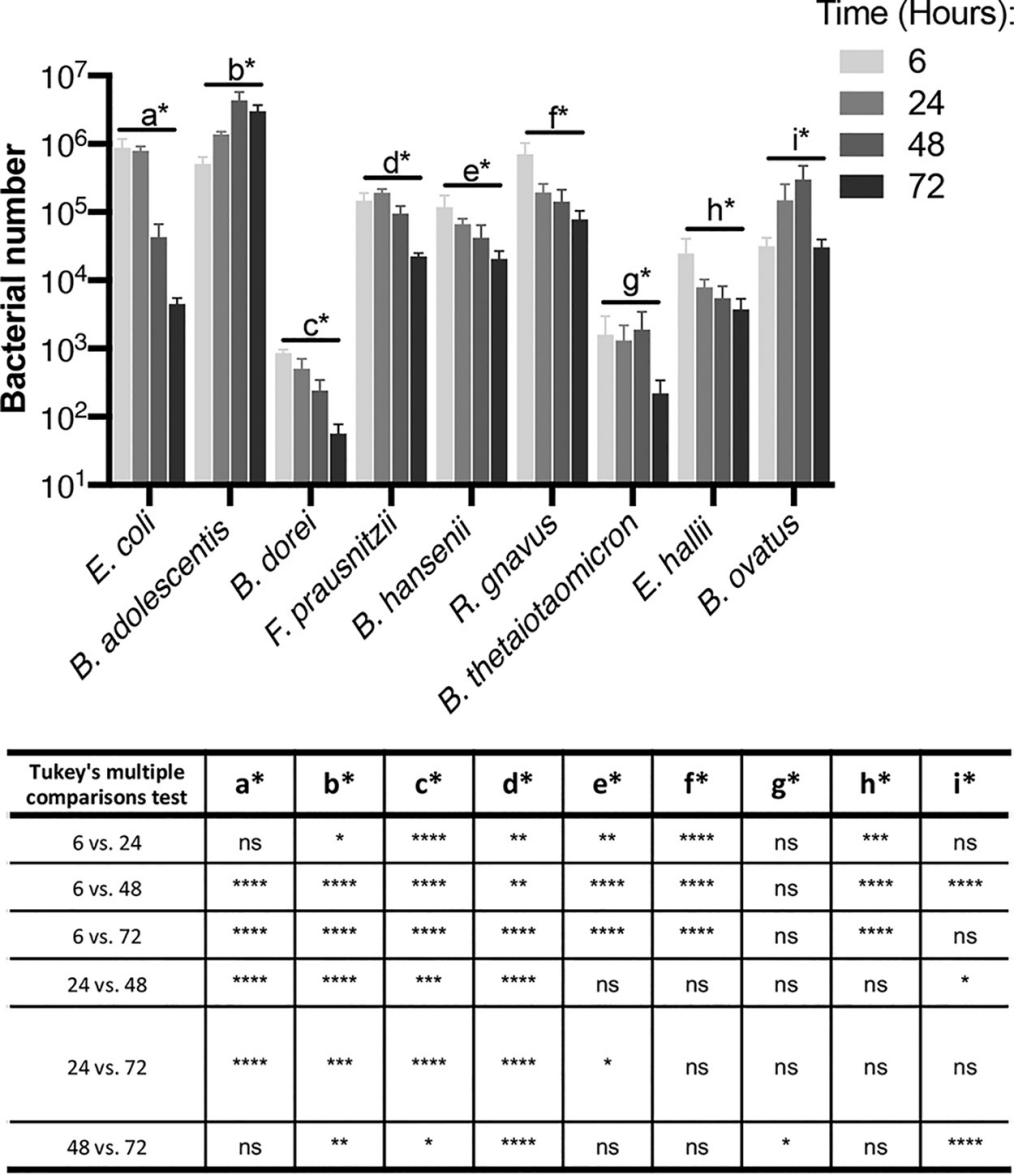
A nine-species gut microbial community tracked over time. Species-specific changes in bacterial numbers within a mixed biofilm were tracked using PMA-qPCR. *C_T_* values were converted using our pipeline to represent total bacterial numbers. Bacterial numbers for individual species within the mixed biofilm at 6, 24, 48, and 72 h are shown in the graph. The table below summarizes the significant differences across time for the different species as calculated by ANOVA, with *post hoc* Tukey’s multiple-comparison test. *P* values: ns, not significant; ****, <0.0001; ***, <0.001; **, <0.01; *, <0.05. Data shown are the mean from three independent biological experiments done in in triplicate. Error bars indicate the standard deviation.

We first tracked numbers of individual species within a mixed biofilm containing nine species. All nine species were detected after different times of incubation up to 72 h (Fig. 2). Bifidobacterium adolescentis and Bifidobacterium ovatus both showed a positive trend at early time points (before 72 h), while B. thetaiotaomicron remained unchanged and all other species showed a reduction in numbers. At 72 h, all the species showed a decay in numbers, implying a buildup of toxic secreted by-products in the medium, or nutritional depletion. Lower bacterial numbers for some species (Bacteroides thetaiotaomicron and Bacteroides dorei) in the inoculum (Fig. S4) (in spite of normalizing numbers by optical density) appeared to impact their numbers within the mixed biofilm. Our results show that species within the biofilm, even within the same genus, showed distinct behaviors, with *Bacteroides* having positive, neutral, and negative trends over the first 48 h.

10.1128/mSphere.00013-21.4 FIG S4 Bacterial numbers of individual species in the mixed inoculum used for mixed-biofilm assays. Bacterial numbers within the inoculum were tracked using PMA-qPCR. *C_T_* values were converted using our pipeline to represent total bacterial numbers. Data shown are the means from three independent biological experiments done in in triplicate. Error bars indicate the standard deviation. Download FIG S4, PDF file, 0.03 MB. Copyright © 2021 Hassall et al. 2021 Hassall et al. https://creativecommons.org/licenses/by/4.0/ This content is distributed under the terms of the Creative Commons Attribution 4.0 International license.

It is worth noting that when the mixed biofilms were compared to the single-bacterial-species biofilms (Fig. S3C), there were significant changes to the numbers of certain community members. An increase of numbers for *B. ovatus* and Blautia hansenii was observed in the mixed biofilms compared with single-biofilm cultures at 24 h, whereas other members like B. thetaiotaomicron, Eubacterium hallii, and E. coli show ∼80-fold, 25-fold, and 20-fold decreases in abundance, respectively, at 24 h. These differences further indicate that individual bacterial dynamics are influenced by interactions within the bacterial community.

Further, we also tested selected pairs of bacterial species to compare how they behaved in dual-species biofilms and the microbiota biofilms. We studied E. coli cultured with selected species which were representative of the different phyla included in the community. For E. coli, the bacterial numbers do not change when incubated with any of the species aside from Ruminococcus gnavus (Fig. S5), which compares well to the unchanged E. coli numbers seen in the mixed community over 24 h (Fig. 2). However, in biofilm cocultures with E. coli, *B. dorei* and *B. hansenii* grew better, while *B. adolescentis* and *R. gnavus* grew less well, and Faecalibacterium prausnitzii remained unchanged, compared to the respective biofilm monocultures (Fig. S5). These growth behaviors are different from those observed in the microbiota community where, for example, *B. hansenii* growth decreases and *B. adolescentis* grows better over 24 h (Fig. 2), Thus, dual-species biofilm interactions are quite distinct from the interactions observed in the microbiota community.

10.1128/mSphere.00013-21.5 FIG S5 Pairwise biofilm analysis of E. coli with 5 species at 24 h. The positive and negative growth interactions were determined by tracking bacterial numbers using PMA-qPCR. *C_T_* values were converted to represent total bacterial numbers. Data shown are the means from three independent biological experiments. Statistical analysis was conducted with a Shapiro-Wilks normality test and Welch’s *t* test. *, *P* value < 0.05; **, *P* value < 0.01. Download FIG S5, PDF file, 0.4 MB. Copyright © 2021 Hassall et al. 2021 Hassall et al. https://creativecommons.org/licenses/by/4.0/ This content is distributed under the terms of the Creative Commons Attribution 4.0 International license.

### Microbiota species interfere in C. difficile adherence and growth.

**FIG 3:**
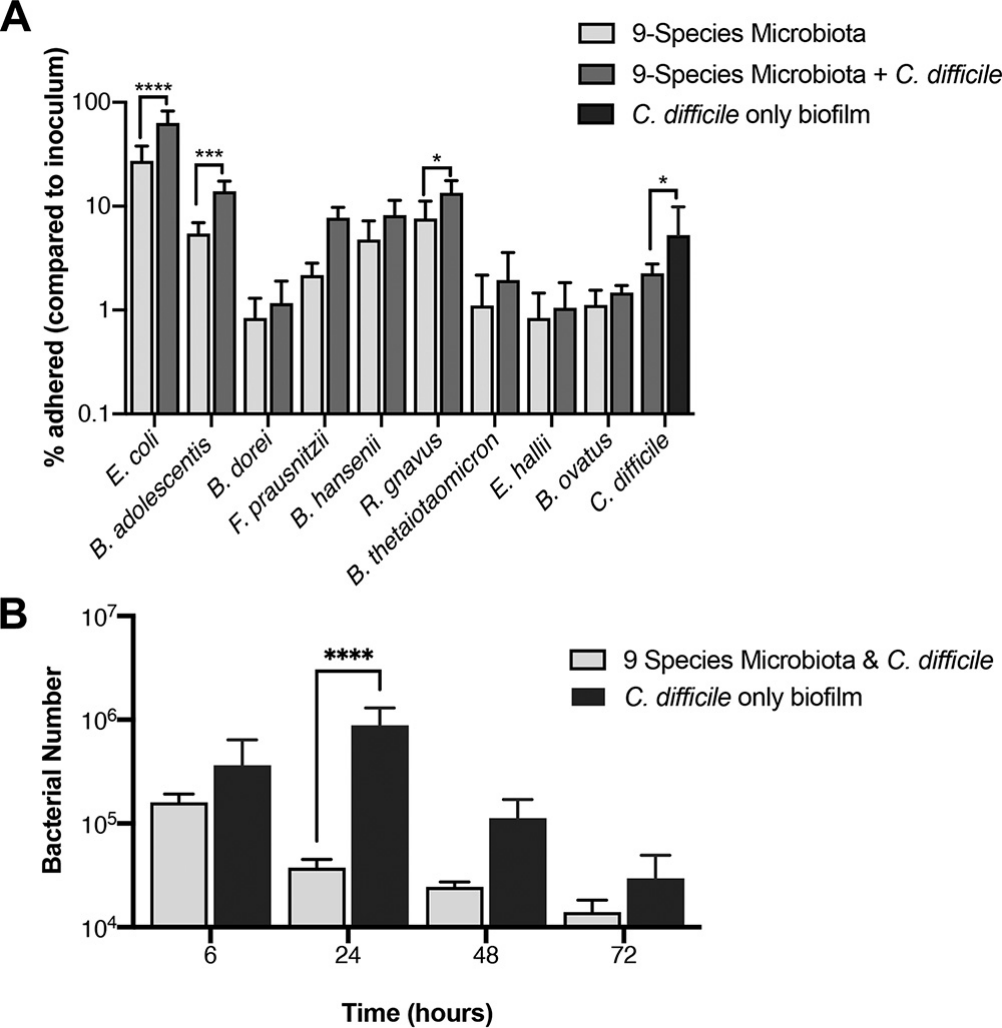
Interactions of C. difficile with a commensal microbiota community. (A) The presence of C. difficile impacts adhesion of several species in the adherent microbiota community. The percentage of inoculum which adhered after 6 h in a nine-species microbiota community (9-species microbiota), in the nine-species community with C. difficile (9-species microbiota + C. difficile), and a single-species C. difficile biofilm control (C. difficile only biofilm). (B) The microbiota has an inhibitory effect on C. difficile. C. difficile bacterial numbers when cultured in monoculture biofilm or with a nine-species representative microbiota were tracked over 72 h using PMA-qPCR. All data shown are means from three independent experiments in triplicate. A two-way ANOVA indicates a significant difference between the two conditions (*P* value < 0.0001), with the *post hoc* Sidak test used to determine specific difference. *P* values: ****, <0.0001; ***, <0.001; *, <0.05.

In order to study the effect of a gut pathogen on the dynamics of this gut microbiota community, we chose to study the effects of the nosocomial pathogen C. difficile. First, to study the effects of the microbiota on C. difficile adhesion, we tracked the formation of adherent biofilms over 6 h in the presence and absence of C. difficile. We measured the percentage of the initial inoculum that is able to adhere to a 24-well polystyrene plate. A significant reduction in initial adhesion was observed in C. difficile when cultured with the microbiota compared to a C. difficile monoculture control (Fig. 3A). Although significant, this reduction is small, with ∼5% of the initial inoculum adhering when cultured alone and ∼2% with the microbiota. With respect to the microbiota, we saw that when cultured alongside C. difficile there was a significant increase in the number of bacteria that adhered. Statistically significant differences were seen for Escherichia coli, *B*. *adolescentis*, and Ruminococcus gnavus, but the trend can be seen generally across all species (Fig. 3A). E. coli appeared to be dominating at this early stage with far more of its original inoculum adhering than any other species. E. coli was the only facultative species present, so any lingering oxygen in the reduced medium may have provided it with an initial head start.

We next investigated the impact on C. difficile during biofilm formation (Fig. 3B). Here, the microbiota impacts C. difficile growth, significantly reducing it at all time points compared to a C. difficile monoculture control. This impact was the highest at 24 h, with the microbiota causing an ∼20-fold drop in C. difficile numbers compared to the monoculture control. Given the higher inhibition seen postadhesion, it is likely that the decrease in C. difficile numbers is attributable to the microbiota negatively impacting C. difficile growth, rather than the reduced ability of C. difficile to adhere.

**FIG 4:**
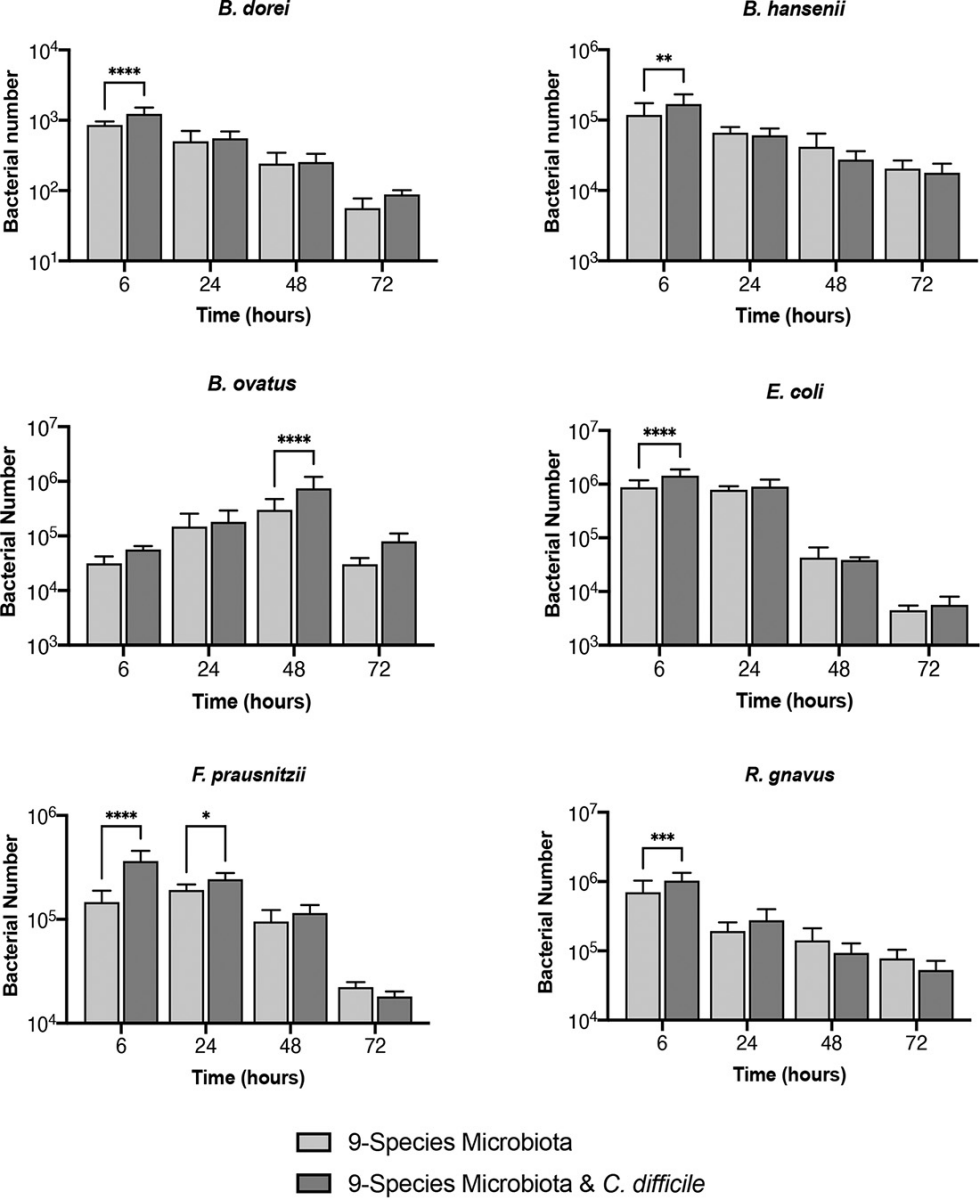
Tracking the effects of C. difficile on individual species within the gut microbiota community. Bacterial numbers of individual species within a representative microbiota and C. difficile biofilm over 72 h tracked using PMA-qPCR are shown compared to a control microbiota biofilm without C. difficile. Data shown are means from three independent experiments in triplicate. A two-way ANOVA was used to determine significant differences between the two conditions, and a *post hoc* Sidak test was used to determine specific difference. *P* values: ****, <0.0001; ***, <0.001; **, <0.01; *, <0.05.

In the presence of C. difficile, we tracked each of the nine species that make up the representative microbiota (Fig. 4 and Fig. S6). When comparing this to a microbiota-only control, we see that the presence of C. difficile has a neutral to positive effect, with six of the nine species having a small but significant difference for *B. dorei*, *B. hansenii*, *B. ovatus*, E. coli, *F. prausnitzii*, and *R. gnavus* at 6 h and/or at 24 h and 48 h. Although C. difficile increases initial binding in the microbiota, the effect is not seen long term. Under both conditions, all species, no matter the prior trajectory, show a decrease in numbers at 72 h, which could be due to the lack of sufficient nutrients in the medium or the accumulation of toxic levels of secreted by-products at this late time point.

10.1128/mSphere.00013-21.6 FIG S6 Effects of C. difficile on individual species from the 9-species microbiota community. Individual species within a representative microbiota and C. difficile biofilm were tracked over 72 h using PMA-qPCR. Data shown are from three independent experiments in triplicate. A two-way ANOVA was used to determine significant differences between the two conditions. Download FIG S6, PDF file, 0.05 MB. Copyright © 2021 Hassall et al. 2021 Hassall et al. https://creativecommons.org/licenses/by/4.0/ This content is distributed under the terms of the Creative Commons Attribution 4.0 International license.

### C. difficile interactions with an established microbiota biofilm.

**FIG 5:**
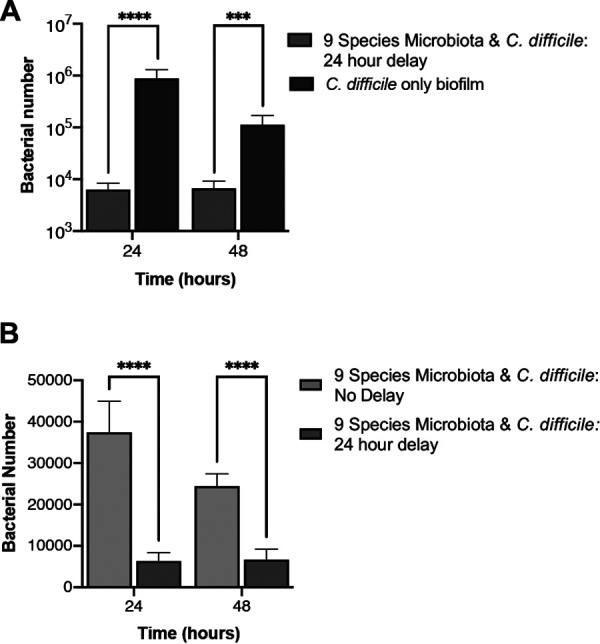
A preestablished microbiota has an inhibitory effect on C. difficile growth. (A) A microbiota biofilm established for 24 h prior to introduction of C. difficile (9-species microbiota + C. difficile 24 h delay) was compared to a C. difficile-only biofilm grown for the same length of time. C. difficile numbers were quantified using PMA-qPCR. (B) The inhibitory effect on C. difficile of preestablishing the microbiota (9-species microbiota + C. difficile: 24-h delay) was compared with the effect on C. difficile cocultured with microbiota from the start (9-species microbiota + C. difficile: no delay). Data shown are means from three independent experiments in triplicate. An unpaired *t* test was used to test for significant difference. *P* value: ****, <0.0001; ***, <0.001.

Usually, the microbiota, when in a healthy state, would already be established before a C. difficile infection. To better replicate this rather than introducing C. difficile at the same time as the microbiota, we preestablished the microbiota biofilm for 24 h prior to introducing C. difficile (Fig. 5A). When comparing this to a C. difficile monoculture grown for the same length of time, we observed that having an established microbiota has a significant inhibitory effect on C. difficile, both 24 h and 48 h postaddition. The largest difference in C. difficile numbers (∼138-fold) was seen at 24 h postaddition. A preestablished microbiota had a significantly larger impact on C. difficile than when the two were seeded together (Fig. 5B). While this is interesting, it is possible that this reduction in C. difficile numbers is not a pathogen-specific effect. To test this, we also studied Bacteroides fragilis, a gut commensal and pathogen, either cocultured together with the microbiota or added to a preestablished community. We observed that B. fragilis numbers are lower when cocultured with the microbiota or added to a preestablished community, compared to a monoculture B. fragilis biofilm. However, when added to a preestablished community, unlike C. difficile, there were higher numbers of B. fragilis (Fig. S7) than when it was seeded together with microbiota. These results may indicate some specificity in pathogen interactions with an established community.

10.1128/mSphere.00013-21.7 FIG S7 Effects of B. fragilis on a preestablished microbiota community. A 24-h preestablished microbiota (9-species microbiota + B. fragilis 24-h delay) has a positive effect on B. fragilis growth at 24 h, compared to B. fragilis cocultured with microbiota (9-species microbiota + B. fragilis no delay), with both conditions showing a decrease in B. fragilis growth compared to monoculture biofilm. B. fragilis numbers were quantified using PMA-qPCR, and *C_T_* values were converted to total bacterial numbers. Data shown are the means from three independent experiments. Statistical analysis was conducted with a Shapiro-Wilks normality test and Welch’s *t* test. **, *P* value < 0.01; ***, *P* value < 0.001. Download FIG S7, PDF file, 0.3 MB. Copyright © 2021 Hassall et al. 2021 Hassall et al. https://creativecommons.org/licenses/by/4.0/ This content is distributed under the terms of the Creative Commons Attribution 4.0 International license.

**FIG 6:**
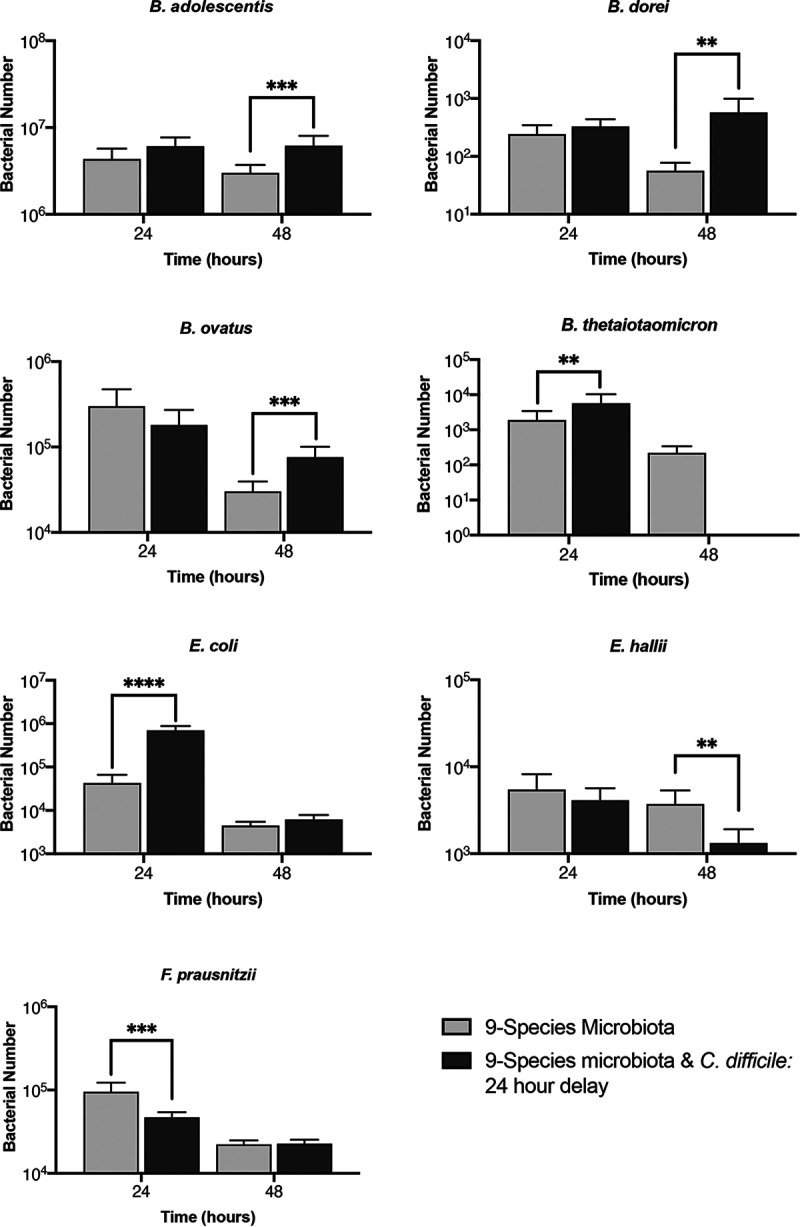
Impact of C. difficile on a preestablished microbiota community. PMA-qPCR was used to track the total number of bacteria for individual species in a nine-species representative microbiota biofilm established 24 h before addition of C. difficile. Data shown are the means from three independent experiments in triplicate. Unpaired Student’s *t* test was used to determine significant differences. *P* values: ****, <0.0001; ***, <0.001; **, <0.01.

We tracked each species in the microbiota to examine any changes in response to C. difficile (Fig. 6). We expected that, as the microbiota was already established, any impact that C. difficile had on the microbiota would be lessened compared to that when the microbiota was seeded with C. difficile. However, surprisingly, we found the opposite to be true. Only two species, *B. hansenii* and *R. gnavus*, did not change in numbers compared to a microbiota-only control, compared to the three seen when C. difficile was added simultaneously. The other seven species all showed significant differences in numbers when C. difficile was introduced (Fig. 6). After 24 h with C. difficile (48 h from microbiota seeding), we saw a positive effect for B. thetaiotaomicron and E. coli. However, we observed a small decrease for *F. prausnitzii*. At 48 h post-infection with C. difficile, we still saw a positive effect on E. coli but to a much lesser degree. An increase was also observed in *B. ovatus*, *B. dorei*, and *B. adolescentis.* Notably, at 48 h, B. thetaiotaomicron undergoes a decrease in numbers compared to the microbiota control, putting it below our detection limit. Thus, although the species impacted by C. difficile between simultaneous culture and addition to preestablished microbiota are distinct, there are several species that are affected under both conditions, including the *Bacteroides* spp.

### Investigating an individual interaction between *Bacteroides* spp. and C. difficile.

**FIG 7:**
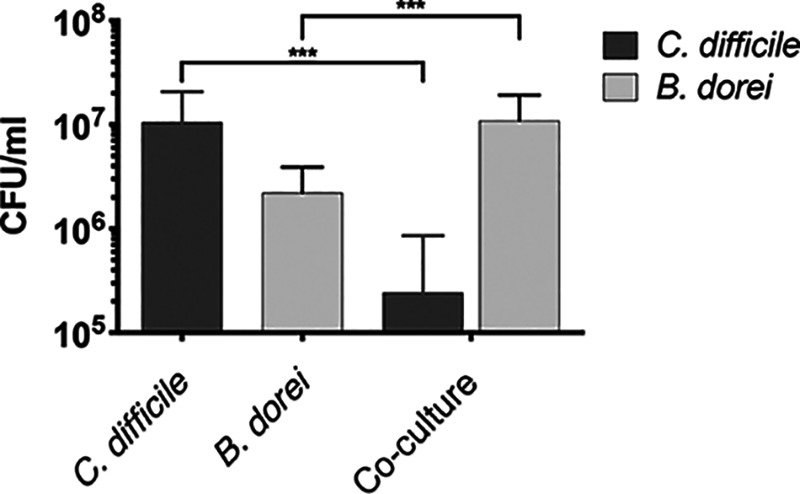
*B. dorei* interactions with C. difficile within biofilms. CFU counts of single or mixed biofilm cultures of *B. dorei* and C. difficile grown for 24 h are shown. Data shown are the means from three independent experiments performed in triplicate, with error bars indicating the standard deviation. Significant difference was determined by an unpaired Student *t* test. ***, *P* value < 0.001.

We wanted to further investigate if species that were increasing in number alongside the C. difficile inhibition were able to affect C. difficile growth. We previously reported that *Bacteroides* spp. multiplies more in the presence of C. difficile when cultured as mixed biofilms (42). We recently studied cocultures of *B. dorei*, an abundant commensal, and C. difficile in the presence of an epithelial cell layer in an *in vitro* system (43) and demonstrated that *B. dorei* multiplies better in the presence of C. difficile, while reducing C. difficile growth. Hence, we investigated inhibitory effects of *B*. *dorei* when cocultured with C. difficile in a dual-culture biofilm. Monocultures and a coculture of the two species were incubated for 24 h, following determination of CFU counts from the biofilms. In the cocultures, we found that the presence of *B. dorei* significantly reduced (by over 10-fold) the number of C. difficile bacteria compared to its monoculture control (Fig. 7). In contrast, *B. dorei*, when cultured with C. difficile, grew far better than when cultured alone. This reduction in C. difficile numbers indicated an inhibition mediated by *B. dorei*. To ensure there was no bias in the initial biofilm inocula, we measured the CFU values of each species; no significant differences were observed between C. difficile and *B. dorei* (Fig. S8A). Interestingly a similar decrease in bacterial numbers was not observed in planktonic culture of both species (Fig. S8B), indicating that the inhibitory effects observed required contact or physical proximity between the two organisms. Our data show that *B. dorei*, one of the commensal *Bacteroide*s spp. that increases within our complex community in response to addition of C. difficile, can negatively impact C. difficile growth.

10.1128/mSphere.00013-21.8 FIG S8 (A) Starting inoculum for C. difficile*-B. dorei* biofilm shows no significant difference when tested with an unpaired Student *t* test. (B) *B. dorei* and C. difficile cultured planktonically as single or mixed cultures for 24 h. Data shown are the means from three independent tests, with error bars indicating the standard deviation. Significant difference was determined by an unpaired Student *t* test; ***, *P* value < 0.001. Download FIG S8, PDF file, 0.02 MB. Copyright © 2021 Hassall et al. 2021 Hassall et al. https://creativecommons.org/licenses/by/4.0/ This content is distributed under the terms of the Creative Commons Attribution 4.0 International license.

## DISCUSSION

Studying molecular interactions between members of the gut microbiota is important in understanding gut homeostasis and important phenomena like colonization resistance. We have developed for the first time a complex mixed-biofilm community comprising nine representative gut commensal species. We report here a PCR-based method for investigating individual interactions within this complex community. We have quantitated changes in bacterial numbers at different times and investigated the impact of the gut pathogen C. difficile on this community. We demonstrate that several species change in numbers in response to addition of C. difficile, including *Bacteroides* spp. We have further demonstrated that one of the *Bacteroides* species, *B. dorei*, can reduce C. difficile growth within dual-species biofilms.

Currently, the predominant techniques used to track interaction between species in the gut are genomics and metagenomics (3, 33–35). Genome sequencing provides excellent insights into what is present but gives no information on short-term dynamics and underlying bacterial interactions. We chose to use a reductionist approach, coculturing a small number of representative gut species so that the output data were more accessible and increased the resolution at which we could measure changes in individual species. Indeed, we have not covered all the key species, but given our chosen quantification technique, it would be possible to now expand this population to include several more species. The main drawback of sequencing is its expense, and hence, it is usually used for analysis of complex samples which contain hundreds of species. Genome sequencing, like standard qPCR, does not eliminate the quantification of “dead” DNA, which is key in accurate quantification of live bacterial numbers. The other alternative to PMA-qPCR is fluorescent *in situ* hybridization (FISH). Several variations of FISH have been used to quantitate bacteria within communities; however, qPCR-based quantitation is generally considered to be more sensitive, more rapid, and less laborious than FISH methods (44–46). Indeed, FISH as a complementary assay would add key spatial details of the community (30). Thus, a PMA-qPCR approach is a simple and reliable method to quantitate changes in individual members of a complex community.

We were able to successfully track all of the species within a nine-species mixed biofilm over a period of 72 h. Interestingly, no single species dominated over others during the experiment; this indicates that rather than direct competition, cross-feeding between species was more likely to be occurring. Henson and Phalak predicted using an *in silico* biofilm metabolic model that a stable cross-feeding relationship between *F. prausnitzii*, B. thetaiotaomicron, and E. coli could be achieved (47). While there are clear trends for each species seen until 48 h, we believe that by 72 h this biofilm is as a whole not stable, with a decline in bacterial numbers observed across all species. This is the case even for species which showed positive signs of growth for the first 48 h (such as *B. ovatus* and *B. adolescentis* [Fig. 2]), and such a decline may be due to the spent medium becoming toxic and/or growth limiting. Indeed, a drawback of static biofilm culture models is the lack of continuous nutrient supply and removal of toxic by-products. However, such models of biofilms are simpler to set up and provide useful information over shorter time frames. Development of such multibacterial biofilms within flow cells which enable continued flow of nutrients under anaerobic conditions would allow monitoring responses for longer periods of time.

Addition of a pathogen to a commensal community would be expected to induce a response to it. To investigate this, studied responses to the gut pathogen C. difficile were introduced. C. difficile is an opportunistic gut pathogen, causing C. difficile infection (CDI), which is the leading cause of hospital-associated diarrhea in the United States, with half a million new cases each year and a repeat infection rate of 1 in 5 patients (48). The best defense against C. difficile colonization is a healthy gut microbiota which provides natural immunity to the disease. Antibiotics negatively affect the gut microbiota alongside the pathogens they are aimed at, resulting in alterations of the gut microbiota and an increased likelihood of CDI (19, 49). Distinct changes in the gut microbiota have been associated with CDI (18); however, as most data are from sequencing of fecal samples, information regarding mucosa-associated populations and changes at early points of colonization is lacking. Interestingly, the presence of C. difficile caused an increase in adhesion for many of the microbiota species within the commensal community (Fig. 3A). We predict either that C. difficile is likely producing a metabolic by-product that improves initial growth and binding for species in the microbiota or that signaling molecules produced by C. difficile are detected by the microbiota and inducing a metabolic shift within these species (50). Further studies using C. difficile are necessary to establish whether soluble factors are involved in the increased adherence observed or whether this is a metabolic effect.

The reduction in the initial adhesion of C. difficile observed when cultured with the microbiota (Fig. 3B) was expected, as there is a wealth of data on the disruptive effect of the microbiota on C. difficile colonization (8). The small (approximately 50%) reduction in adherence may be because the microbiota community is not preestablished, which is normally the case for an infecting C. difficile. Bile acids and salts have been shown to have a considerable impact on the ability of C. difficile to colonize the gut, with the microbiota converting the primary bile acids required for C. difficile germination such as taurocholic acid (21, 51). The secondary bile acids into which they are converted can also inhibit C. difficile growth (23). There were no bile acids in the medium that we use, suggesting that the C. difficile inhibition observed was not through the conversion of primary to secondary bile acids. Hence, the prevention of bacterial germination through secondary bile acid production appears to be only part of the mechanism for colonization resistance. Indeed, when the microbiota biofilm was preestablished 24 h prior to introducing C. difficile, we found that C. difficile growth was substantially impacted (Fig. 5A and B). Preestablishing the microbiota could result in increased abundance of the commensal bacteria and result in higher levels of any secreted inhibitory molecules. Additionally, physical space required for C. difficile to adhere would be far less and any nutrients required by C. difficile could already be taken out of the medium by this stage.

In a successful infection, C. difficile has been reported to control the microbiota by modulating bacterial metabolism, including the production of indole (52, 53). It does this through influencing the expression of tryptophanase (*tnaA*) in other species; C. difficile is thought to limit the recovery of the microbiota through indole-mediated inhibition of growth of protective gut bacteria (53). Small but significant increases in the numbers of *B. dorei*, *B. ovatus*, E. coli, *F. prausnitzii*, and *R. gnavus* were observed when C. difficile was cocultured with the commensal community. Although of the nine species, *B. ovatus*, B. thetaiotaomicron, *B. adolescentis*, E. coli, and *F. prausnitzii* are indole producers (53–56), no inhibitory effects were seen. Given that C. difficile was instead inhibited, it is possible that the bacteria were unable to reach sufficient numbers to influence indole production. Also, after the addition of C. difficile to a preestablished community, we saw an increase in E. coli numbers compared to the control (Fig. 6). Again, an overabundance of *Proteobacteria* was often found in CDI patients and was a characteristic of a successful C. difficile infection (20, 57). However, the increase in numbers of multiple *Bacteroides* spp. that was observed in parallel may explain the inhibition of C. difficile observed in this system. C. difficile infections have been associated with a significant decrease in *Bacteroidetes*, which may suggest a protective role for these bacteria in the gut (57, 58).

Interestingly, C. difficile growth is negatively impacted when cocultured with Bacteroides dorei, an abundant gut commensal species (Fig. 7). A reduction in C. difficile growth in the presence of Bacteroides fragilis was reported previously (42). Notably, B. fragilis, much like *B. dorei*, had higher numbers in mixed culture with C. difficile than it did on its own, and growth-inhibitory effects were specific to biofilm growth, indicating that cell-to-cell interactions/physical proximity may play a role. We also recently reported that C. difficile growth on epithelial cells in an *in vitro* gut model was reduced in the presence of *B. dorei* (43). B. fragilis has been recently reported to prevent C. difficile infection in a murine infection model by potentially impacting the integrity of the epithelial barrier (59). While these data support a role for *Bacteroides* spp. in preventing C. difficile infection, patients infected with C. difficile generally have a reduction in the abundance and diversity of *Bacteroidetes* (57). Our data may suggest that without a prior microbiota disturbance, for example with antibiotic treatment, C. difficile is unable to bring about a decrease in *Bacteroidetes* numbers but instead reinforces *Bacteroidetes* dominance by improving growth.

In summary, we report a very useful *in vitro* tool that could be used to assess behaviors of members of a complex microbial community. We have used this microbiota model to show the inhibitory effects on C. difficile and the changes triggered by it in specific microbiota species. This model enables easy tracking of dynamic changes in the microbiota in response to C. difficile and other pathogens. Further studies on the molecular changes in this microbiota community using “omics” technologies like transcriptomics will reveal new mechanisms involved in resisting C. difficile. Indeed, this community can be expanded or changed to include other representative species and host cell components (for example, gut epithelial cells) and used to track transcriptomic and metabolic changes modulated in response to stress factors including drugs and pathogens.

## MATERIALS AND METHODS

### Bacterial culture.

All the bacterial strains listed in Table 1 were cultured at 37°C under anaerobic conditions using an anaerobic cabinet (Don Whitley DG250), and unless stated otherwise, cultures were grown in Schaelder anaerobic broth (SAB; Oxoid) supplemented with 0.005 mg/ml vitamin K (VWR) and 2 mg/ml l-cysteine (Sigma-Aldrich) (here called SAB+).

### Microbiota biofilm assay.

For mixed biofilms with multiple microbiota species, individual bacterial cultures were grown in SAB+ for 16 to 18 h (overnight at 37°C in an anaerobic cabinet). These cultures were then diluted with fresh SAB+ to achieve a final concentration of 0.1 optical density at 600 nm (OD_600_) per species within the mixed culture. To ensure consistency in experiments with differing numbers of species (with and without C. difficile), we supplemented the absence of a species with an equivalent amount of medium, i.e., under each condition the concentration of the conserved species remained the same. The diluted cultures were added together and inverted several times to ensure a homogenous mix. One milliliter of culture mixture was added per well of a 24-well polystyrene tissue culture-treated dish, and biofilms were allowed to form at 37°C for the required time (6 h to 72 h) in an anaerobic cabinet. At each time point, the wells were gently washed twice with 1 ml phosphate-buffered saline (PBS) and resuspended in PBS. The resuspended biofilms were then treated with PMA, after which a DNA extraction was conducted.

### Propidium monoazide treatment.

Prior to DNA extraction, propidium monoazide (PMA; Biotium) was added to the resuspended samples at a final concentration of 40 μM. The samples were then incubated in the dark for 10 min (37°C, in an anaerobic cabinet). To photoactivate the PMA, samples were activated in the PhAST Blue (GenIUL) light system for 15 min, following which genomic DNA was extracted.

### Genomic DNA extraction.

DNA extraction was carried out using a phenol chloroform-based method. Cultures were centrifuged at 14,000 rpm for 5 min, after which the supernatant was discarded. The cell pellets were resuspended in 500 μl of 5 mg/ml lysozyme (VWR) and incubated for 20 min at 37°C. Following this, 20 μl RNase solution (20 mg/ml) (Fisher Scientific), 20 μl proteinase K (20 mg/ml) (New England Biolabs), and 25 μl sodium dodecyl sulfate (SDS) (Fisher Scientific) were added. The samples were then incubated at 37°C for a further 10 min; following this, 100 μl of NaCl (Fisher Scientific) and 80 μl cetyltrimethylammonium bromide (CTAB) (Sigma-Aldrich) were added before a final incubation at 60°C for 45 min.

The lysed samples were treated with 750 μl of phenol-chloroform-isoamyl alcohol (PCI) (Sigma-Aldrich), vortexed (2 to 3 s), and centrifuged for 10 min at 14,000 rpm. The upper phase was transferred to a fresh Eppendorf tube, and the PCI centrifuge steps were repeated until a clear boundary could be seen between the two phases. Next, 75 μl of 3 M sodium acetate (pH 5.2) (Sigma-Aldrich) and 750 μl of cold (−20°C) 96% ethanol were added. This solution was inverted until the DNA precipitated out. The precipitated DNA solutions were then centrifuged for 5 min at 14,000 rpm. The pellets were washed with 200 μl 70% ethanol and centrifuged for a final time (2 min at 14,000 rpm). The DNA pellet was dried at room temperature and resuspended in 75 μl Tris-EDTA (TE) buffer.

### Real-time quantitative PCR (qPCR).

Primers were designed to target either the topoisomerase I (*topI*) or DNA gyrase subunit A (*gyrA*) region of each strain (Table 2). The primers were designed using Primer-BLAST (60, 61), to generate amplicons of 100 to 150 bp. All primers had an annealing temperature of 59 to 60°C, were screened for target sequence specificity against the “nt” database, and had low probability for formation of possible primer-dimer structures as determined by Thermo Fisher’s multiple primer analyzer. The qPCR was conducted with the Agilent Mx3005P qPCR system and the Luna universal qPCR master mix (New England Biolabs).

**TABLE 2:** Primer sequences used in quantification

Species	Gene of interest	Sequences (5′ to 3′)
*B. dorei*	*topI*	Forward: AAGCGGCTTCAAGAAACAGG; reverse: GTGCCCTTTACCTTGGGAAC
B. fragilis	*gyrA*	Forward: GTGCCCTTCCCGATGTTAGA; reverse: TCCGTGCGGGTGATACTTAC
*B. ovatus*	*topI*	Forward: GGGCCTATTATCGCAACCGA; reverse: AGGTGCATACGTAGACGGAC
B. thetaiotaomicron	*topI*	Forward: GTCTGTAATCAAGTCCGCCG; reverse: AATGCCGGAAAGCGGTAAAC
*B. adolescentis*	*topI*	Forward: CTCCGGATACACGGTCATGG; reverse: GTCTTCGATATCCACGCCGA
*B. hansenii*	*gyrA*	Forward: GACGTAAGAAGCACCGGTAGA; reverse: ATAATCGCCCTGACAGGTAAGC
C. difficile	*gyrA*	Forward: GGTTGAAAGAATAGCAGAGTTAGTT; reverse: GCATTAGCATCCCTCTTTAATTCTA
E. coli	*gyrA*	Forward: GAACTCGGTGAGGACGGTTT; reverse: GCTGGAACAGGACGAACGTA
*E. hallii*	*gyrA*	Forward: TACCGCCTCATCGGACTTGA; reverse: TCATGGAGGCTGGATGCTCT
*F. prausnitzii*	*gyrA*	Forward: CCGGTGTCCGTGTCATGC; reverse: CTCAGCCTCTACTGTCTCGG
*R. gnavus*	*gyrA*	Forward: GCTGAACAGAGCAGAAGAGC; reverse: TCCTTCGCAGTCTGAACATTCT

To convert from a qPCR cycle threshold (*C_T_*) to a predicted bacterial number, we utilized previously described methods (61, 62). For each species, *C_T_* values were plotted against DNA concentrations, as quantified using a Qubit fluorometer 2.0 (Thermo Fisher) and a dsDNA Qubit kit (Thermo Fisher). A semilog line was fitted to each curve, with a regression above 0.990 (see Table S1A and B in the supplemental material). These standard curves were used to convert *C_T_* values into the starting mass of DNA (*M*_DNA_) (equation 1). Using the assumption that one genome weight worth of DNA is equal to one bacterium, total bacterial numbers were calculated by dividing the starting amount of DNA by the calculated genome mass (*M*_Genome_) (equation 2). *M*_Genome_ was calculated by multiplying the length of the genome (*G*_Length_) by the average weight of 1 bp (*W*_Base_), where *W*_Base_ = average weight of 1 mole (650 Da)/Avogadro’s number (*N_A_*) (Table S2).

(1) $$M_{\text{DNA}}=10^{\frac{C_{T}-m}{c}}$$

where *m* is gradient, *c* is intercept, and *C_T_* is the cycle threshold value.

(2) $$\text{Predicted bacterial number}\approx \text{number of genomes}=M_{\text{DNA}}\left(\frac{1}{M_{\text{Genome}}}\right)=M_{\text{DNA}}\left(\frac{N_{A}}{G_{\text{Length}}W_{\text{Base}}}\right)$$

10.1128/mSphere.00013-21.10 TABLE S2 Genome length, weight, and accession numbers of the sequences used for PMA-qPCR quantification conversion into bacterial number. Download Table S2, PDF file, 0.03 MB. Copyright © 2021 Hassall et al. 2021 Hassall et al. https://creativecommons.org/licenses/by/4.0/ This content is distributed under the terms of the Creative Commons Attribution 4.0 International license.

### C. difficile and *B. dorei* biofilm studies.

*B. dorei* and C. difficile were cultured overnight in brain heart infusion (BHI) medium (Sigma-Aldrich) supplemented with 0.5 mg/ml yeast extract (Fisher Scientific) and 0.001 mg/ml l-cysteine (BHIS) (43). After this, cultures were diluted down to an optical density (OD) of 0.1 and added to a 24-well polystyrene tissue culture-treated plate. Each well was made up to a total of 1 ml with monocultures having a mix of 0.5 ml culture and 0.5 ml BHIS medium and cocultures containing 0.5 ml of each species. The resulting end concentration was an OD at 600 nm (OD_600_) of 0.05. At set time points, the biofilms were washed twice with 1 ml PBS and then manually resuspended in 1 ml PBS. Dilutions were plated on BHIS with C. difficile supplement (Oxoid). C. difficile and *B. dorei* have two distinct colony morphologies (43), which allowed us to differentiate between *Bacteroides* and C. difficile and quantitate each species.

### Statistical analysis.

All experiments were performed in triplicates and repeated at least three times independently. An unpaired Student’s *t* test was used to determine if differences between two groups were significant, and a two-way ANOVA was used to compare multiple groups.
