# Erratum for Hassall et al., “Dissecting Individual Interactions between Pathogenic and Commensal Bacteria within a Multispecies Gut Microbial Community”

**DOI:** 10.1128/msphere.00893-24

**Published:** 2024-11-21

**Authors:** Jack Hassall, Jeffery K. J. Cheng, Meera Unnikrishnan

## ERRATUM

Volume 6, no. 2, e00013-21, 2021, https://doi.org/10.1128/msphere.00013-21. Page 13: Equation 1 should read as follows.

$$M_{DNA}=10^{\frac{CT-c}{m}}$$

