# Supplementary figures and images for: Dissecting Individual Interactions between Pathogenic and Commensal Bacteria within a Multispecies Gut Microbial Community
Source: mSphere. 2021 Mar 24;6(2):e00013-21. doi: 10.1128/mSphere.00013-21 (PMC8546675; doi:10.1128/mSphere.00013-21)

# Suppl Figure 1

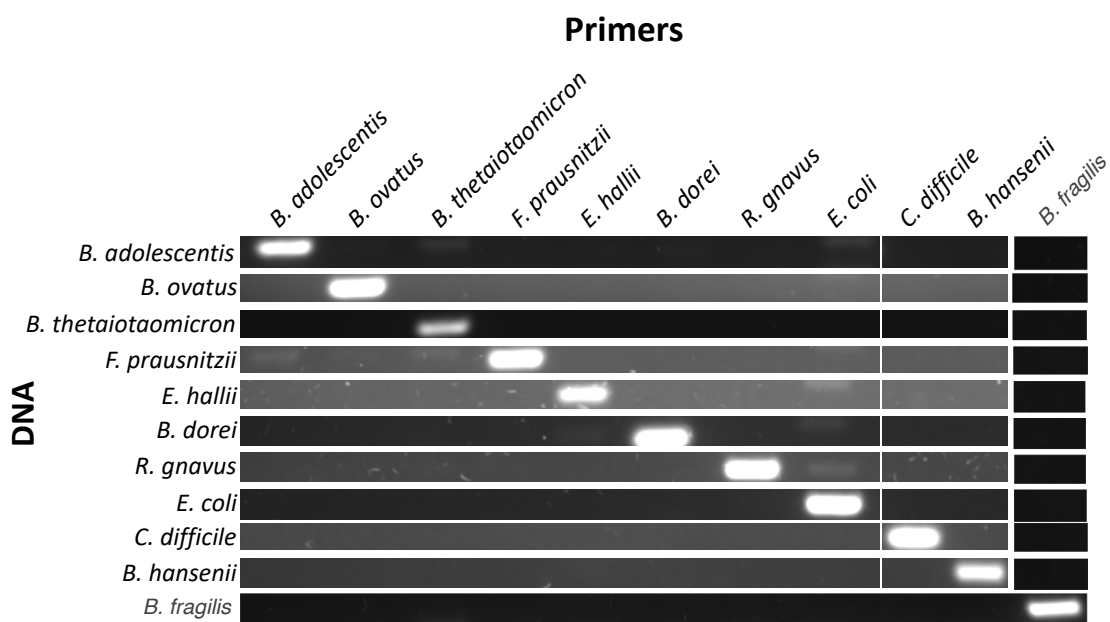

Supplement: FIG S1 [file msphere.00013-21-sf001.pdf]

Suppl Figure 2

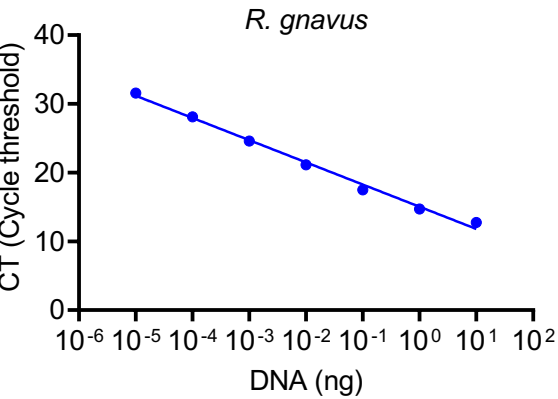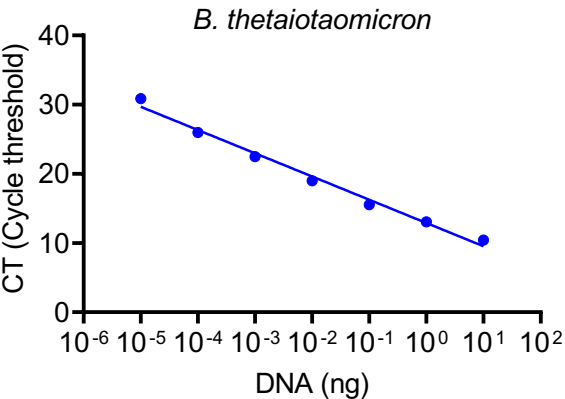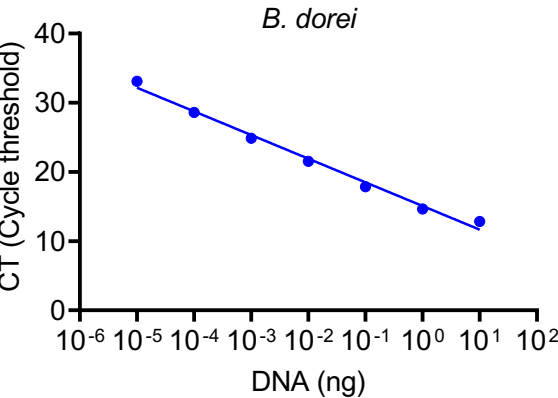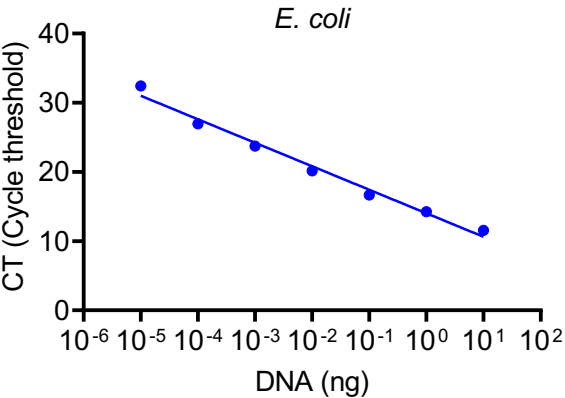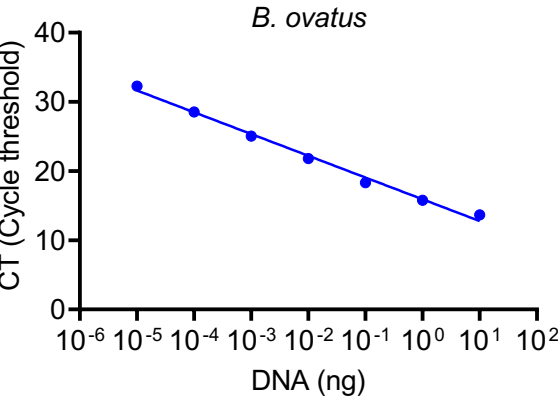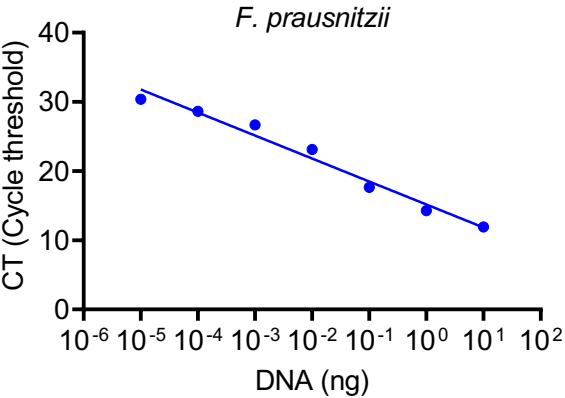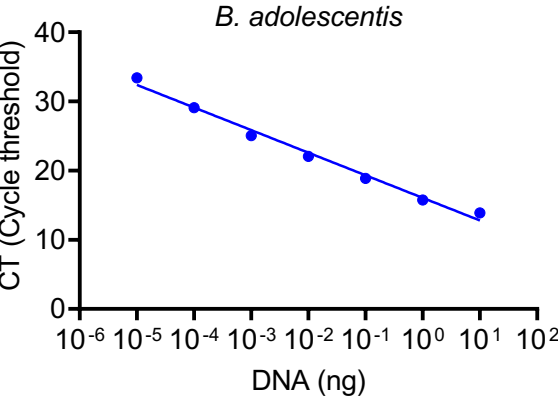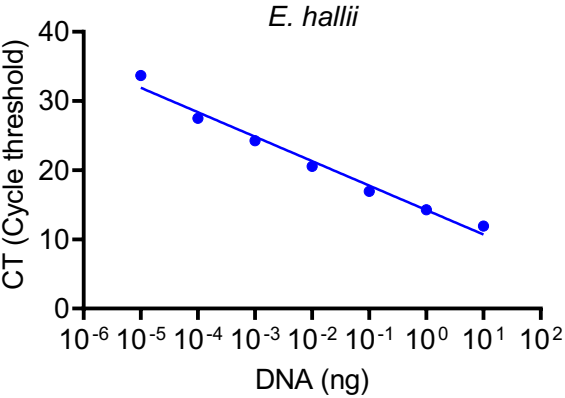

Suppl Figure 2 (continued)

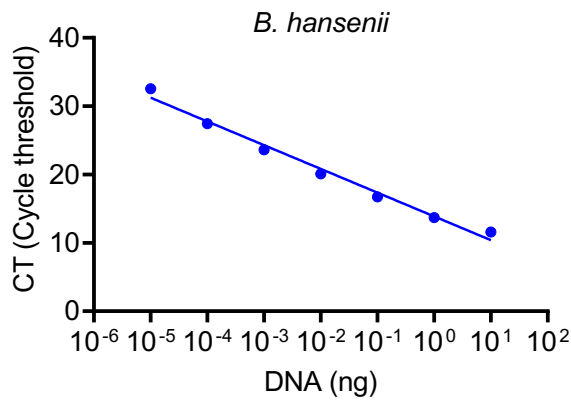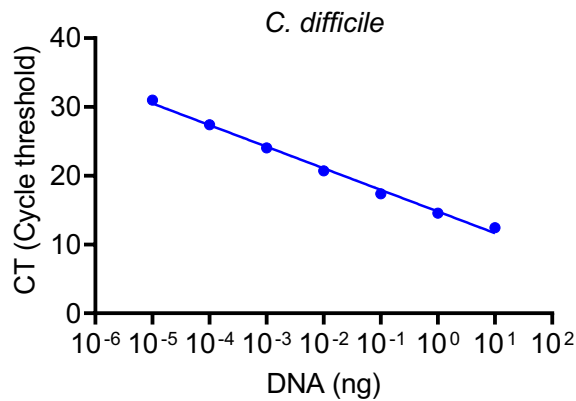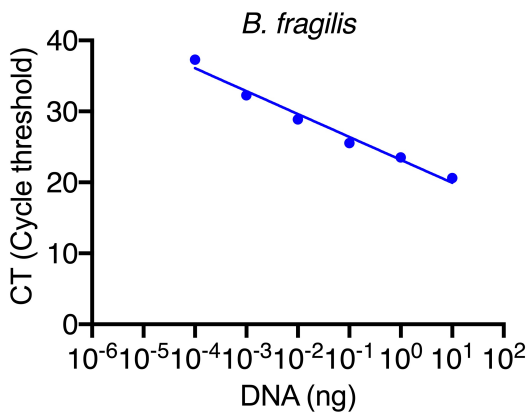

Supplement: FIG S2 [file msphere.00013-21-sf002.pdf]

Suppl Figure 3

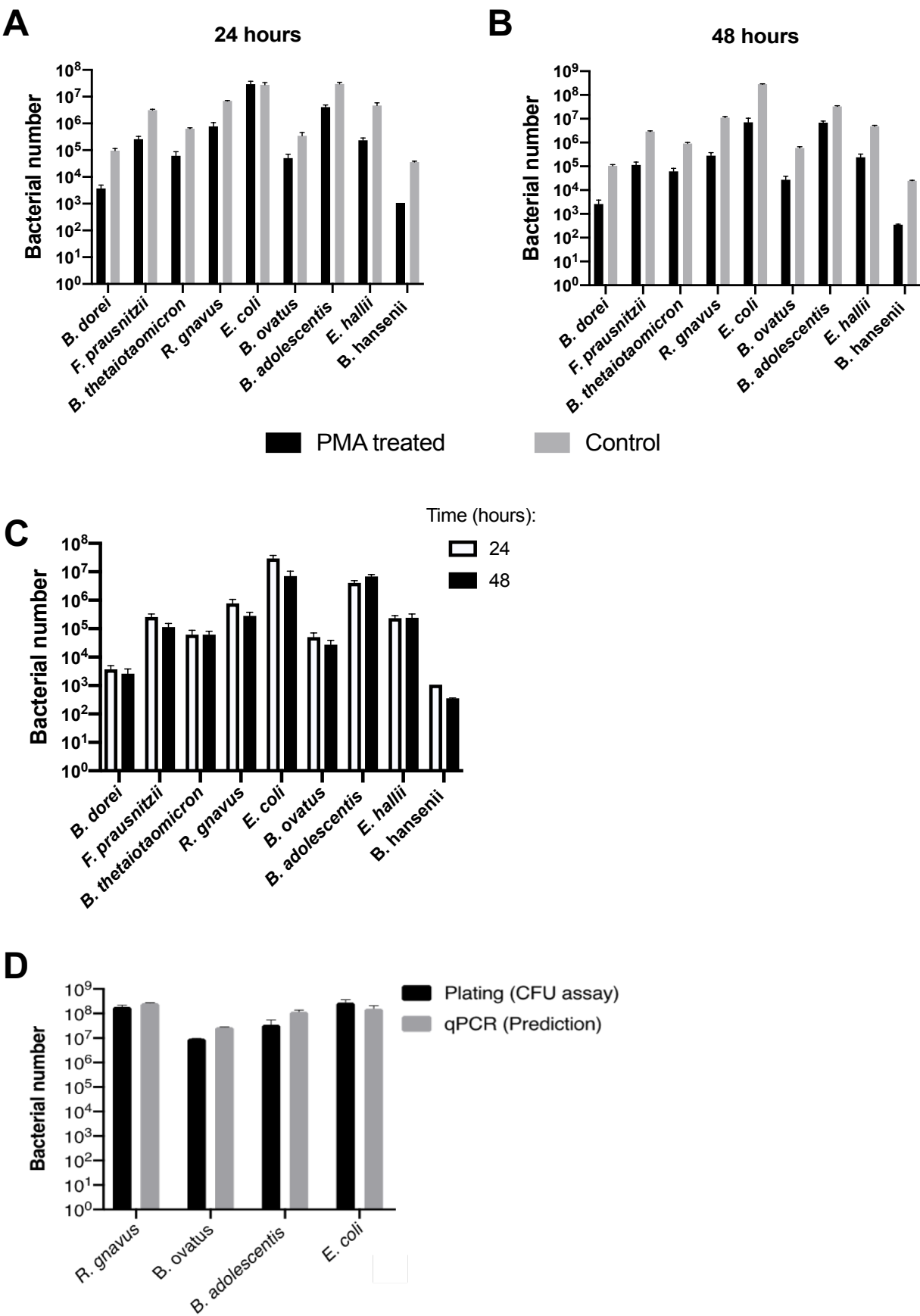

Supplement: FIG S3 [file msphere.00013-21-sf003.pdf]

Suppl Figure 4

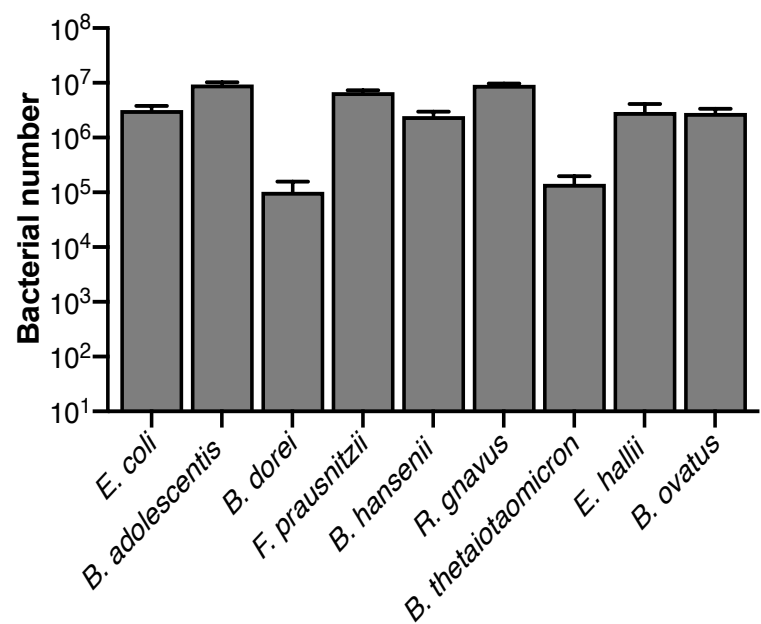

Supplement: FIG S4 [file msphere.00013-21-sf004.pdf]

Suppl Figure 5

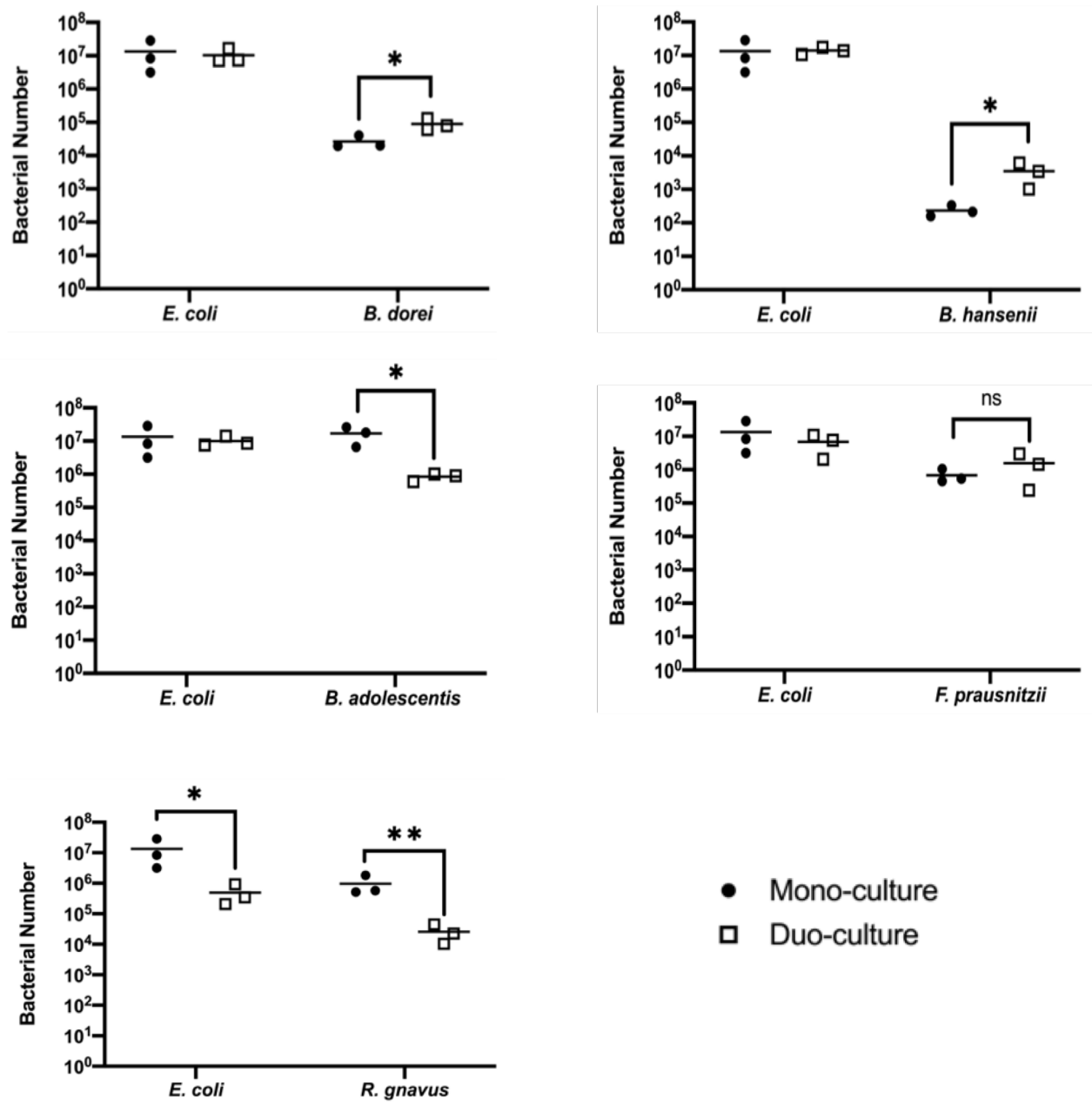

Supplement: FIG S5 [file msphere.00013-21-sf005.pdf]

Suppl Figure 6

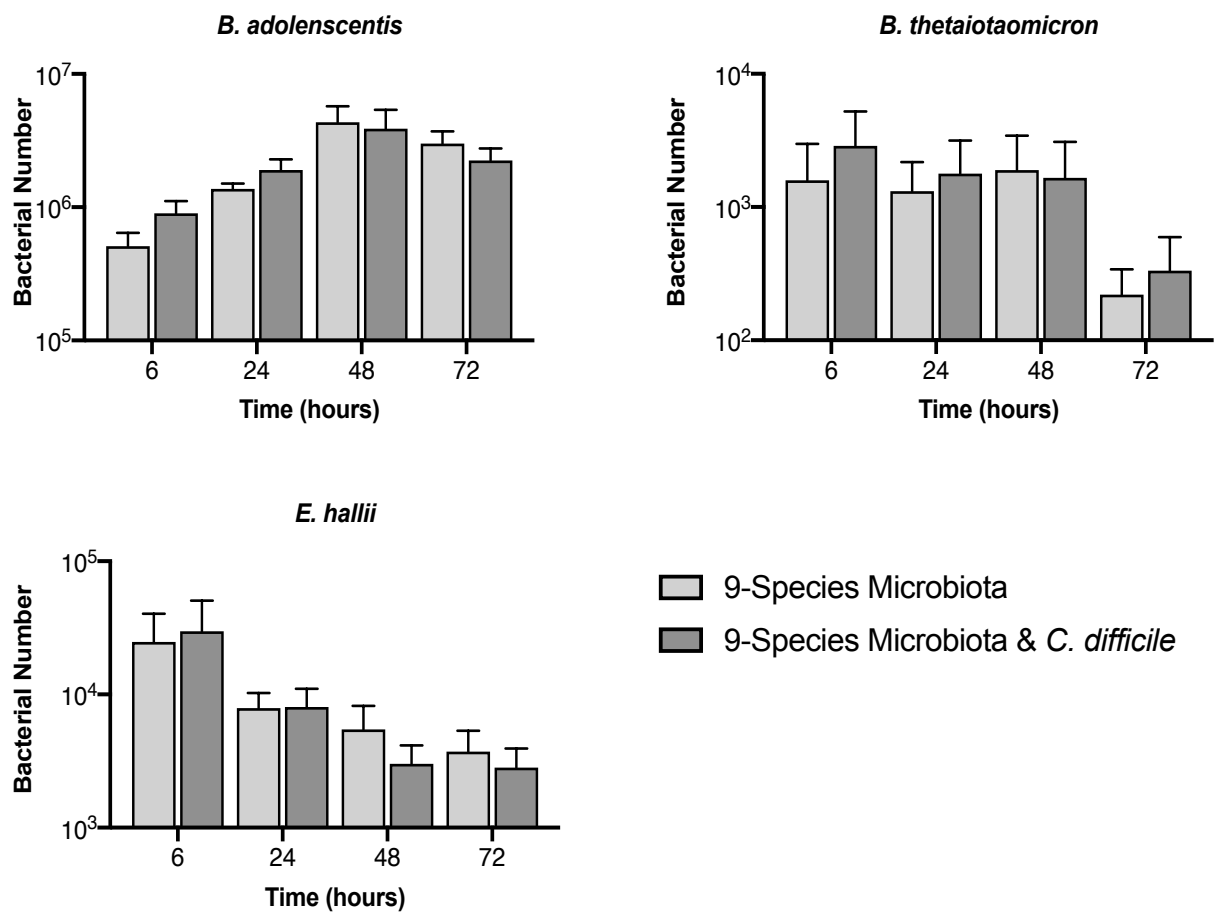

Supplement: FIG S6 [file msphere.00013-21-sf006.pdf]

Suppl Figure 7

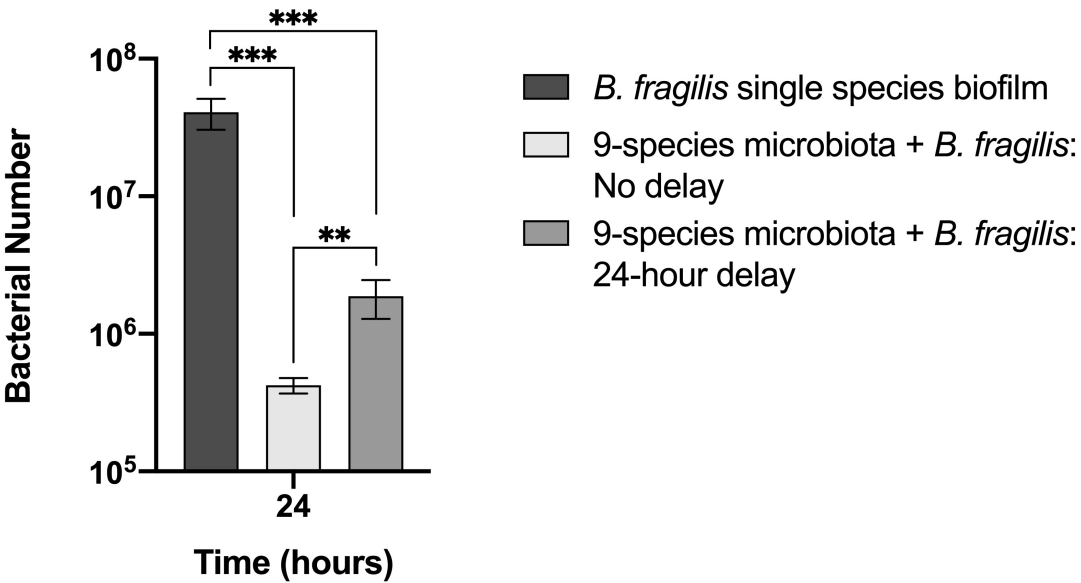

Supplement: FIG S7 [file msphere.00013-21-sf007.pdf]

Suppl Figure 8

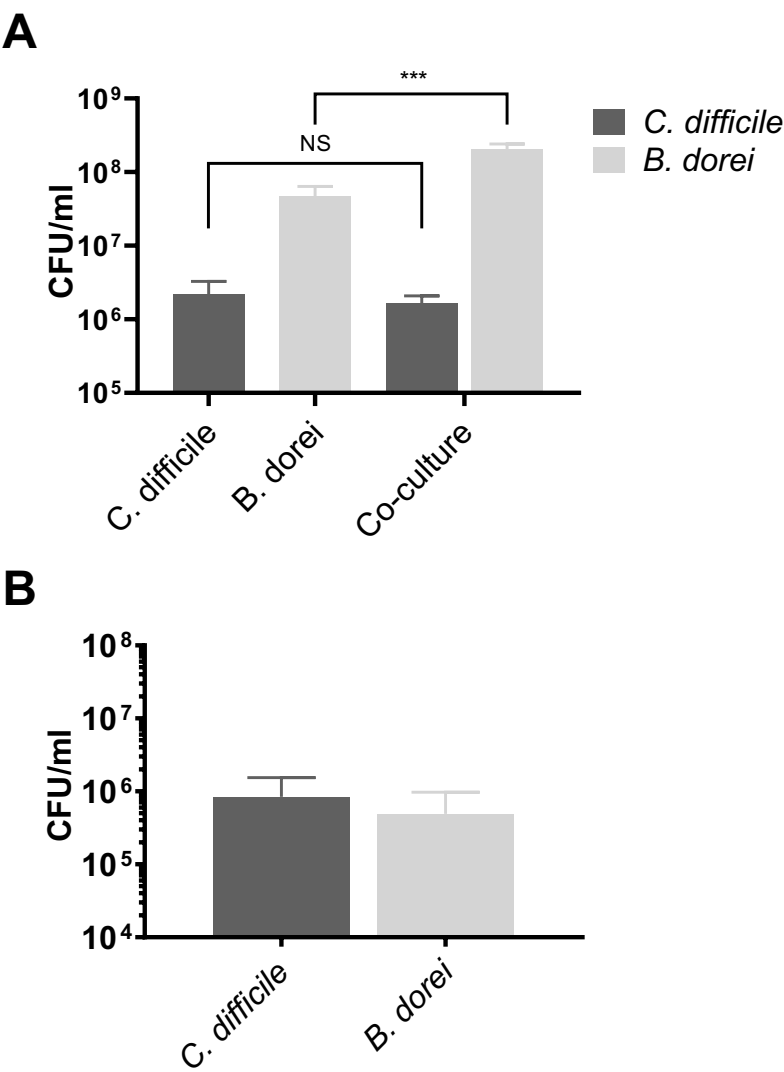

Supplement: FIG S8 [file msphere.00013-21-sf008.pdf]
