# Supplementary material for: Dissecting Individual Interactions between Pathogenic and Commensal Bacteria within a Multispecies Gut Microbial Community
Source: mSphere. 2021 Mar 24;6(2):e00013-21. doi: 10.1128/mSphere.00013-21 (PMC8546675; doi:10.1128/mSphere.00013-21)
Supplement: TABLE S1 [file msphere.00013-21-st001.pdf]

Table S1A

Species	Slope	Y-intercept	R^2	Primer efficiency (%)
B. dorei	-3.269	14.26	0.9994	102.26
B. fragilis	-3.230	22.20	0.9919	107.90
B. ovatus	-3.224	14.74	0.9994	104.26
B. thetaiotaomicron	-3.396	14.91	0.9992	97.00
B. adolescentis	-3.858	18.57	0.9944	81.64
B. hansenii	-3.522	13.03	0.9951	92.28
C. difficile	-3.651	14.56	0.9991	87.89
E. coli	-3.450	16.73	0.9986	94.92
E. hallii	-3.545	18.61	0.9903	91.46
F. prausnitzii	-3.171	17.01	0.9927	106.71
R. gnavus	-3.263	15.98	0.9979	102.52

Table S1B

Species	Slope	Y-intercept	R^2	Primer efficiency (%)
B. dorei	-3.419	15.08	0.9888	96.10
B. fragilis	-3.228	23.18	0.9777	104.07
B. ovatus	-3.146	15.91	0.9919	107.90
B. thetaiotaomicron	-3.361	12.89	0.9890	98.39
B. adolescentis	-3.266	16.05	0.9861	102.39
B. hansenii	-3.475	13.87	0.9852	93.99
C. difficile	-3.143	14.79	0.9945	108.05
E. coli	-3.398	14.02	0.9822	96.92
E. hallii	-3.536	14.23	0.9778	91.78
F. prausnitzii	-3.324	15.16	0.9586	99.91
R. gnavus	-3.230	15.03	0.9912	103.98
