# Supplementary material for: Dissecting Individual Interactions between Pathogenic and Commensal Bacteria within a Multispecies Gut Microbial Community
Source: mSphere. 2021 Mar 24;6(2):e00013-21. doi: 10.1128/mSphere.00013-21 (PMC8546675; doi:10.1128/mSphere.00013-21)
Supplement: TABLE S2 [file msphere.00013-21-st002.pdf]

Table S2

Species	Length (b)	Weight (ng)	NCBI Reference
B. dorei	6079576	6.56×10^{-6}	GCF_000273035.1
B. fragilis	5530115	5.97×10^{-6}	GCF_000025985.1
B. ovatus	6549476	7.07×10^{-6}	GCF_000218325.1
B. thetaiotaomicron	6293399	6.79×10^{-6}	GCF_000011065.1
B. adolescentis	2389110	2.58×10^{-6}	GCF_000154085.1
B. hansenii	3065949	3.31×10^{-6}	GCF_002222595.2
C. difficile	4191339	4.52×10^{-6}	GCF_000027105.1
E. coli	5106156	5.51×10^{-6}	GCF_000159295.1
E. hallii	3290996	3.55×10^{-6}	GCF_000173975.1
F. prausnitzii	3090349	3.34×10^{-6}	GCF_000162015.1
R. gnavus	3181861	3.43×10^{-6}	GCF_000507805.1
